# Electric clothes dryers: An underestimated source of microfiber pollution

**DOI:** 10.1371/journal.pone.0239165

**Published:** 2020-10-07

**Authors:** Kirsten J. Kapp, Rachael Z. Miller

**Affiliations:** 1 Department of Arts and Sciences, Central Wyoming College, Jackson, WY, United States of America; 2 Rozalia Project for a Clean Ocean, Burlington, VT, United States of America; VIT University, INDIA

## Abstract

Microplastics, particularly microfibers, are ubiquitous, found in aquatic (freshwater and marine) and terrestrial environments and within the food web worldwide. It is well-established that microplastics in the form of textile fibers enter the environment via washing machines and wastewater treatment effluent. Less is known about the release of microfibers from electric clothes dryers. In this study we measure microfiber emissions from home installed dryers at two different sites. At each site the distribution of fibers landing on the snow’s surface outside dryer vents and the weight of lint in dryer exhaust exiting dryer vents were measured. Fibers from the pink polyester fleece blankets used in this study were found in plots throughout a 30ft (9.14m) radius from the dryer vents, with an average number across all plots of 404 ± 192 (SD) (Site 1) and 1,169 ± 606 (SD) (Site 2). The majority of the fibers collected were located within 5 ft (1.52m) of the vents. Averages of 35 ± 16(SD)mg (Site 1) and 70 ± 77 (SD)mg (Site 2) of lint from three consecutive dry cycles were collected from dryer vent exhaust. This study establishes that electric clothes dryers emit masses of microfiber directly into the environment. Microfiber emissions vary based on dryer type, age, vent installation and lint trap characteristics. Therefore, dryers should be included in discussions when considering strategies, policies and innovations to prevent and mitigate microfiber pollution.

## Introduction

Microplastic pollution, particularly in the form of microfibers, is an issue of growing concern for both human and environmental health, and is identified as one of the key environmental challenges of our time. Global plastic production increased by 29% between 2011 and 2018 (average annual increase of 3.7%), and a total of 359 million metric tons were produced in 2018 [1, 2]. However, these figures do not include the majority of fibers in textile production, which in 2018 reached 107 million metric tons, of which approximately 62% were synthetic (such as polyester, polyamide etc) [3].

When microplastics enter the environment, they have the potential to harm organisms and disrupt ecosystem processes. Measuring the toxicity of microplastics to our organisms and environment is complex [4] and inconclusive. Research on the impacts of microplastics on organisms and our environment does not show similar results in part due to different polymers (i.e. polyethylene, polystyrene, PVC, PET etc), different shapes (i.e. spheres, fragments, fibers etc), different organisms (i.e annelids, crustaceans, molluscs etc), and varying doses and exposure time used in the experiments [4]. Nonetheless, in a review of toxicological studies, it is reported that more studies measuring the effects of microfibers, in particular, showed some sort of negative impact on the organism studied than those that did not [4]. For example, microfibers had longer gut retention times and observed slower growth rates in *Hyalella azteca* when compared to the effects of ingesting microplastic particles [5]. In zooplankton (*Ceriodaphnia dubia*) microfibers had more deleterious effects when exposed to both microfibers and polyethylene beads [6]; however, the negative effects did not result from ingestion (no fibers were detected in the organism’s gut) but rather from physical damage to external body parts such as their antennae and carapace caused by microfibers.

An abundance of microfibers have been found in aquatic (freshwater and marine) and terrestrial environments worldwide [7–11]. Recent studies have observed microplastics, the majority of which were microfibers, in the French Pyrenees, the Italian Alps and in snow samples from the Arctic [12, 13]. While these studies suggest that atmospheric transport may be an important source of microplastic pollution in otherwise pristine areas far and wide, little is understood about the sources of airborne microfibers, their transport and deposition [8, 14].

It is well understood that the shedding of microfibers from clothing during wash cycles introduces thousands of microfibers into the environment via washing machine effluent. For example, it is estimated that over 6,000,000 fibers are potentially released from a 5kg wash consisting solely of polyester fabrics [15]. The number and weight of fibers released during wash cycles under various conditions has been studied many times [16–21]. Because of a lack of research, perhaps due to assumptions that lint traps effectively stop fibers from entering the environment, little is known about the release of microfibers from electric clothes dryers [19] and to date, the effectiveness of dryer lint traps in retaining microfibers.

In the United States, 80.3% of households use a clothes dryer (94.9 million households) as of 2015 [22]. US households use their dryers for an average of 439 cycles per year [23]. In 2009, 90.2 million households used a dryer, 82% (74.4 million) used them every time clothes were washed, while 15% (13.8 million) used them for some but not all loads and only 2% (2.1 million) households use their dryer infrequently [24]. While Canada has similar dryer usage to the US (81% in 2009) [25] dryer usage varies worldwide. Outside of the US and Canada, there is significantly less dryer usage and it varies by country (Table 1).

**Table 1:** Household ownership of electric clothes dryers in select countries.

Country	% households with a dryer	Year	Source
Australia	55%	2014	Australian Bureau of Statistics [26]
Canada	81%	2009	Statistica [25]
France	38%	2011	Schmitz and Stamminger [27]
Germany	42%	2017	Statistica [28]
Japan	34.6% dryer only		Brasur and Stubuku [29]
	50% combined		Gooiger and Stamminger [30]
	washer/dryer (although 92% report not using dry function)		
Libya	18% (a 12% increase from 2012)	2013	Mohamed et al [31]
Norway	47%	2012	Statistics Norway [32]
Sweden	52%	2011	Schmitz and Stamminger [27]
United Kingdom	58%	2018	Statistica [33]

In addition to the US and Canada usage numbers, as developing nations see an increase in the middle class and access to less expensive power, the dryer market is growing. For example, while air drying clothes has traditionally been preferred in Asian countries, with an increasing standard of living and pollution concerns, dryer sales have seen an increase [34]. Although little data on the number of households owning dryers exist, sales increased by more than 100% year-over-year in 2019 [35]. In 2019 the global market for electric dryers was $7.3 billion [36]. It is estimated that the global market for electric dryers will reach $10.8 billion in 2023 [36] and $13.4 billion in 2024 [37].

Due to the potential for microfibers, both synthetic and non-synthetic, to cause ecological harm, upstream mitigation efforts are underway to reduce their input into the environment such as products that specifically address microfiber pollution by capturing fibers during the laundry wash cycle (in-drum and external devices) [38]), and strategies that address the greater problem of microplastic in our public waterways such as bans on plastic microbeads in personal hygiene products, single use plastic bans, efforts to improve waste infrastructure and management practices, consumer education campaigns and developing alternative compostable or biodegradable materials. As of yet, no mitigation strategies are in place for electric clothes dryers. However, the dryer usage and growth numbers above indicate that dryers, particularly in the US and Canada, but also worldwide could contribute a large volume of microfibers to the environment that require them to be part of the discussion of microfiber pollution.

The objectives of this study are to: 1) Measure the amount of fibers (by weight) emitted via dryer vent exhaust, 2) map the distribution of fibers released that settle onto the ground at varying distances from the dryer vent using a polyester fleece blanket and 3) establish that differences in dryer types, ages, vent installation and lint trap characteristics may produce different amounts of fiber emissions. Results from this study can help inform policy, drive further research and innovation and assist in developing strategies that ultimately lead to a reduction in global microfiber pollution.

## Methods

### Study area

**Fig 1:**
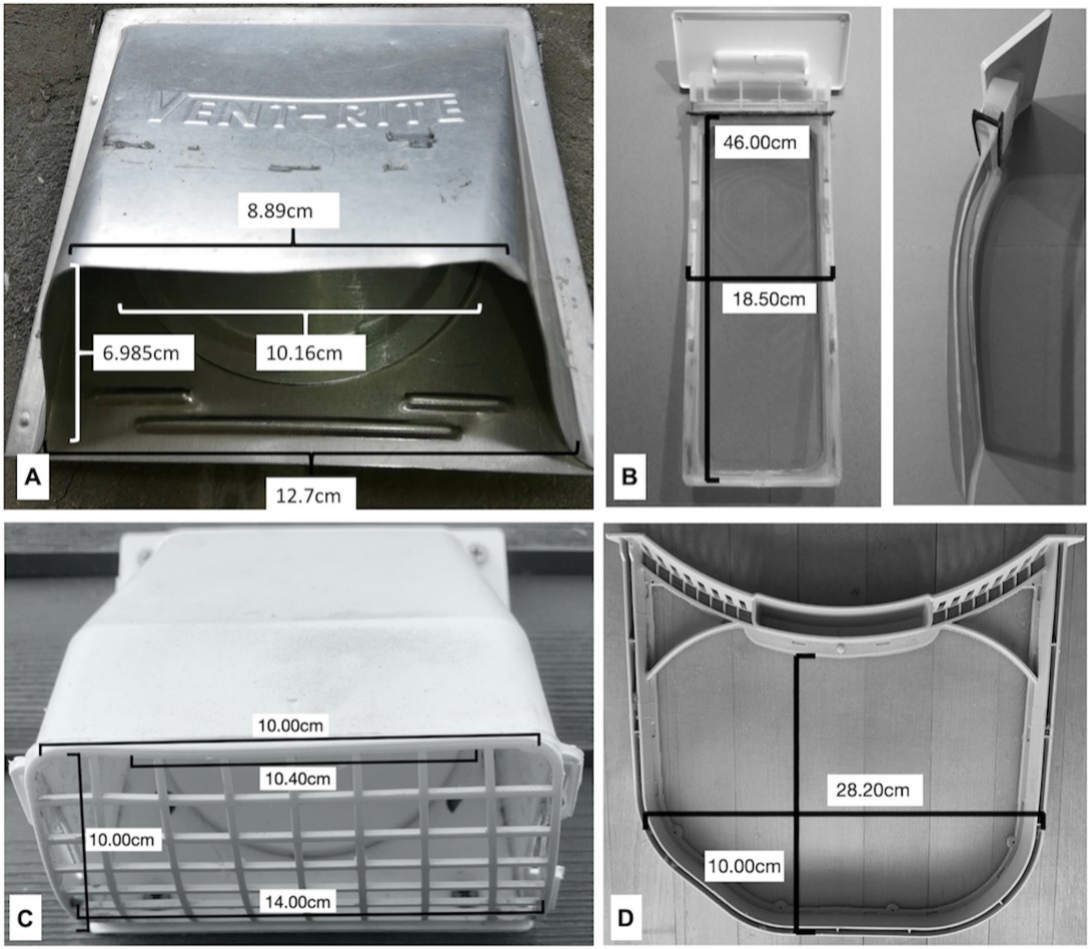
Images taken of the dryer vents and lint traps used in this study showing vent and lint trap screen dimensions for the electric clothes dryers at Site 1 (A and B) and Site 2 (C and D).

In order to best represent real-world conditions, experimentation was carried out in homes at two locations, Idaho and Vermont (from here on referred to as Site 1 and Site 2, respectively), using two different domestic dryers. Site 1, located at 6200’ elevation, was equipped with a ROPER electric dryer (model RED4640YQ1), which was purchased new between 2008 and 2009. The lint trap dimensions are 18.5cm by 46cm with 1mm^2^ screen mesh size. The vent (Vent-Rite) faces southeast and is 0.997m from the ground. It has a damper (diameter = 10.16cm) but no screen (Fig 1). The length of the rigid ducting is 6m.

Site 2, located at 1500’ elevation, was equipped with a LG TrueSteam 7.4 Cu. Ft. 12-Cycle Electric Dryer (LG model DLEX3570W), which was new in October 2018. The lint trap dimensions are 28.2cm x 22.5cm with 1mm^2^ screen mesh size. The dryer is against the wall through which the vent is placed 1.397m from the ground facing East. The length of the semi-rigid ducting is 1.2m. The vent is a trapezoid shape with a damper (diameter = 10.40cm) and screen (openings 1cm x 1.2–1.5cm) (Fig 1).

### Materials

Twelve identical 100% polyester 127cm x 152 cm fleece blankets (438 ± 17g) were used in this study (Golden Linens LLC Ultra Soft Cozy Plush Fleece Traveling Throw Blanket), one for each replicate. The blankets were hot pink, as this was not a color used or worn in either household and would be easy to detect on white filters. Snow samples were collected using metal or wood rulers, metal spoons and glass jars. Melted snow samples were vacuum filtered using Nitrocellulose Mixed Ester (MCE) membrane Filters (5μm, 47mm). Dryer vent exhaust was sampled using 100 μm white nylon mesh filter bags (Duda Energy LLC).

### Blanket preparation

Due to high variation of fiber shedding caused by washing machines and because this study was meant to isolate fiber emissions from electric clothes dryers and not that of washing machines, washing machines were not used first. One new blanket was removed from its original packaging and submerged (not agitated or stirred in any way) in a 5-gallon bucket of cold water for five minutes. The wet blanket was then removed and repeatedly compressed (not twisted or wrung) against the side of a tub until very little water came out. Finally, the blanket was hung over a drying rack, the edges compressed to get the last of the water out and left to drip for 30 minutes (S1 File). The blanket was then placed in the electric clothes dryer on the automatic dry low heat setting for approximately 1 hour.

### Surface snow sampling: Fiber count

**Fig 2:**
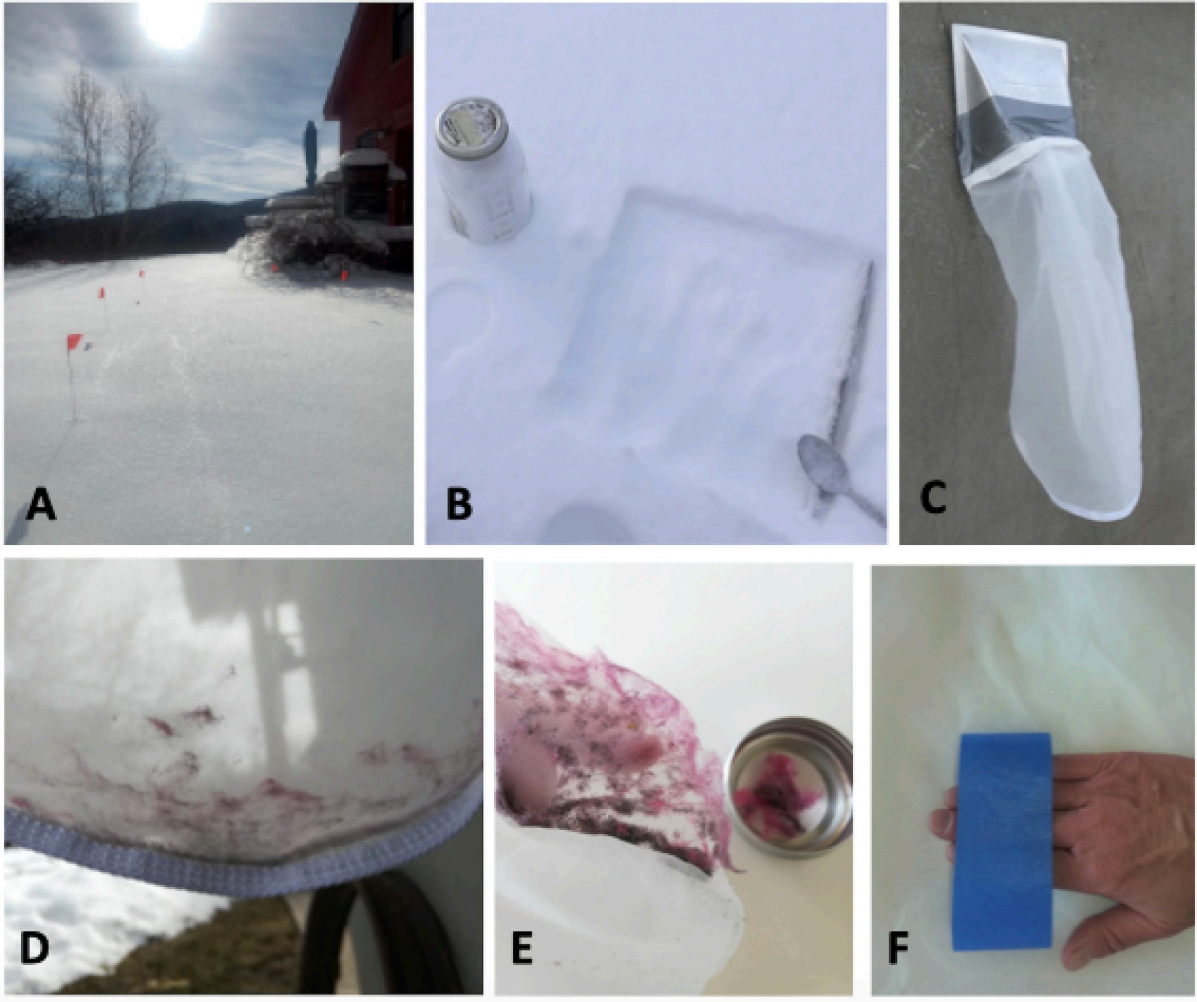
Methods used in this study. A-B) Surface snow sample collection, C) Dryer vent exhaust collection D) Example of lint captured in bag during dryer cycle, E) Removal of lint from bag by hand, and F) Removal of remaining fibers on bag with tape.

**Fig 3:**
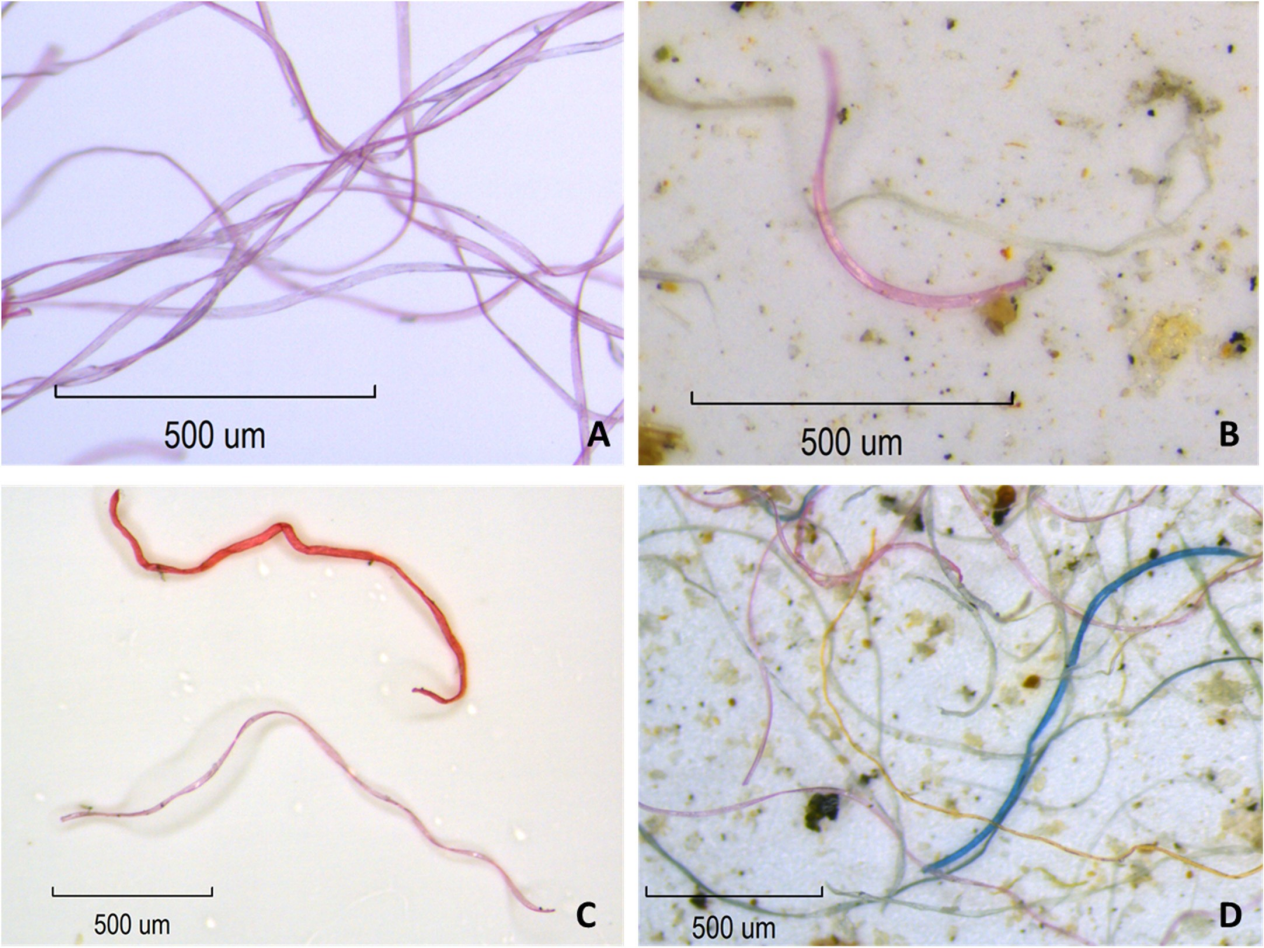
A) Example of pink polyester fibers collected from fleece blanket used in this study, B) Example of pink fiber from fleece blanket found in snow sample, C) Comparison between pink fiber from fleece blanket and darker pink fiber identified as “other” and D) Example of fibers observed in snow sample.

**Fig 4:**
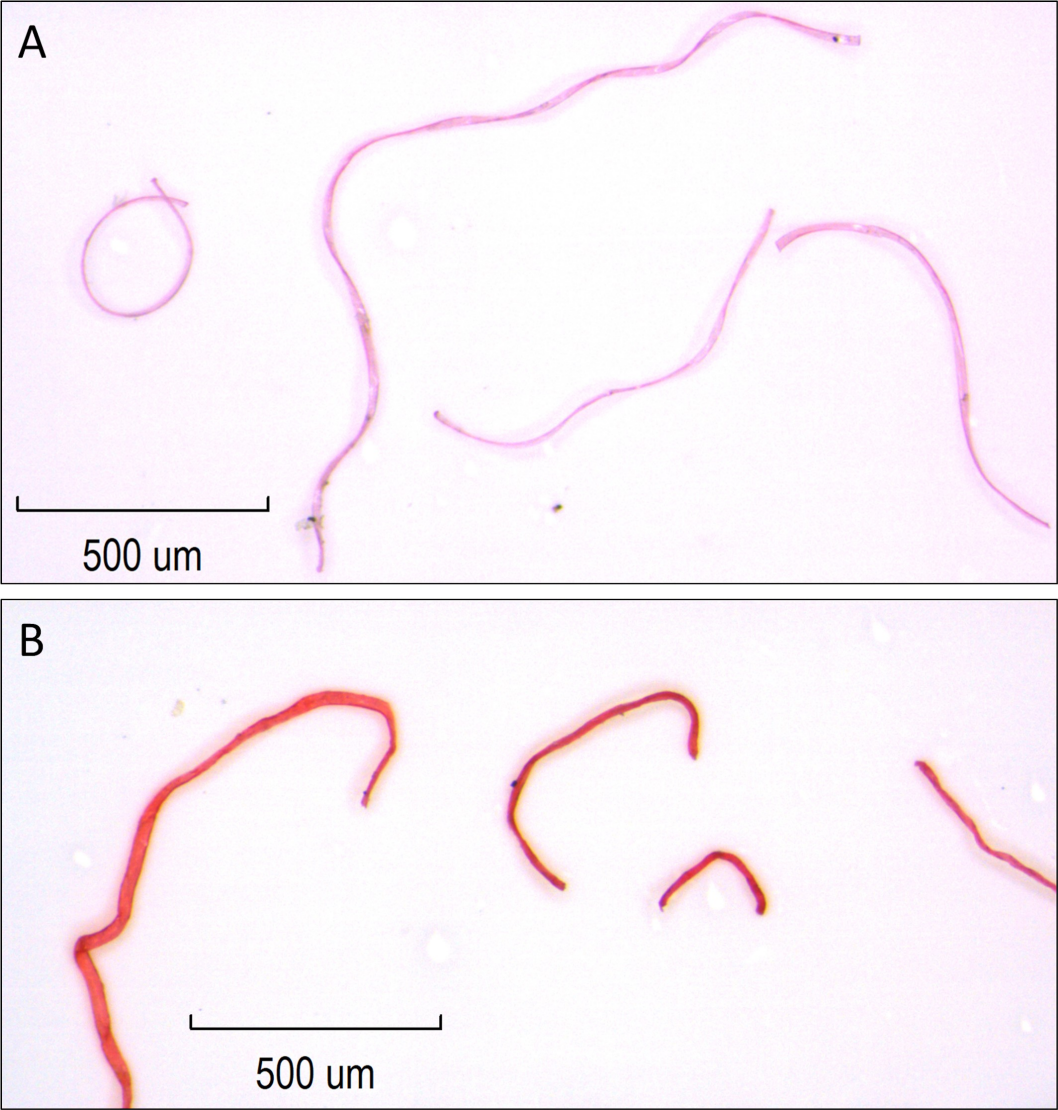
A comparison of the fibers extracted from the pink fleece blanket used in this study (A) and darker pink fibers observed in tap water and snow samples identified as “other” (B).

Immediately after the dry cycle was complete, surface snow samples (top 2.54cm) were collected from 0.093m^2^ plots at 5ft (1.52m), 10ft (3.05m), and 15ft (4.57m) intervals directly perpendicular from the dryer vent and 5ft (1.52m) on either side (Fig 2A and S1 File). An additional 5 samples were collected at 30 ft (9.14m) in an arc from the vent (resulting in a total of 14 surface snow samples per sampling event). A new blanket was used for each sampling event (n = 3 per site for a total of n = 6), which were completed after a fresh snowfall (at least 7.62cm) on days with a wind forecast of less than 5 mph (2.24 m/s). At each of the plots, a 0.093m^2^ area was marked with a stainless steel or wooden ruler (Fig 2B). Using a stainless steel spoon, the top 2.54cm of surface snow was collected from inside the square and packed into a 1.89L (Site 1) or into two 1L (Site 2) glass mason jars (Fig 2B). Once melted, snow samples were filtered (5 μm Nitrocellulose Mixed Ester (MCE), 47mm) using a vacuum filtration system. Each filter was immediately placed into a clean aluminum or glass dish, covered, and dried at room temperature. Once dry, all fibers were counted. Based on visual characteristics, the pink fibers from the blanket were easily identified using a stereoscope (Figs 3 and 4). Other anthropogenic textile fibers not from the pink experimental blanket were identified by morphology (e.g uniform thickness, no branching, smooth margins etc) [8, 39] and color (e.g. dark pink, blue, red, clear, purple) and recorded as “other”. Fibers that were straight, had irregular edges or margins (i.e. not equally thick along the entire length and/or exhibited structures such as nodes or branches) were eliminated. In cases of doubt, fibers were not considered to be anthropogenic and were excluded from the count. While other pink fibers were observed in the samples, they were distinguished from blanket fibers by color and morphology (Fig 4) and counted as “other”.

Due to the ease of identifying fibers from the pink polyester blankets and differentiating them from other anthropogenic fibers, as well as the large numbers of fibers observed in this study and the associated costs, additional methods such as FTIR or Raman microscopy were not used to identify the chemical composition of fibers observed in the samples. Furthermore, the types of “other” fibers observed (e.g. nylon, cotton, wool, cellulose etc) varies with each household, and determining the numbers of each type was not an objective of this study.

### Dryer vent exhaust: Weight

Total fiber output was collected by duct taping a 100 μm nylon mesh bag to the outside vent (Fig 2C). This size mesh was selected as the smallest mesh size least likely to affect airflow. A flow meter (Testo 417) was used to confirm the 100 μm mesh bags did not disrupt the airflow blowing from the vent. The same blanket preparation and drying methods above were performed prior to sample collection. The dryer was run empty for one whole cycle, then dryer lint traps and outside vents were cleaned prior to each sampling. Due to subtle changes in humidity causing weight variation of empty nylon bags, it was determined that the most accurate way to weigh the lint captured in dryer exhaust was to remove it from the bag and weigh it separately. First, all visible lint was removed by hand (Fig 2E), placed in an aluminum tin and dried in a drying oven at 50°C for at least 8 hours, after which they were weighed three times (draft free Ohaus Pioneer PA163,0.001mg). Next, a pre-weighed piece of tape (3M Scotch Blue) was used to remove any remaining fibers (similar to a lint roller) and weighed again (Fig 2F). Therefore, the total weight of lint collected from each bag is the sum of lint removed by hand and by tape. After thorough cleaning, each bag was inspected under a stereoscope (Nikon SMZ800N, magnification 15x - 120x) to ensure only a negligible amount of fibers remained, if any. The lint from lint traps was removed from each dryer, dried and weighed as per methods above.

### QA/QC

To reduce procedural contamination, snow samples were only collected after snowfalls of more than 3in (7.62cm). Dryers were run empty for a full cycle before each new snowfall and each new sampling event in order to reduce/eliminate capturing fibers leftover from earlier regular household dryer cycles. Dryers were not used after a new snowfall and prior to a sampling event. All glassware was rinsed three times with tap water and tap control blanks were taken on all days that tap water was used. None of the tap blanks (n = 7) contained pink fibers resembling those from the fleece blanket, however some contained dark pink and other colored fibers (Site 1 = 2 ± 2(SD)/L; Site 2 = 7.5 ± 2.5(SD)/L). During vacuum filtration, the filtration apparatus was covered with a watch glass to reduce air exposure and only filtered water (5 μm Nitrocellulose Mixed Ester (MCE), 47mm) was used. Only black and grey clothes were worn during outdoor snow sample collection, and white cotton lab coats were worn during sample processing. Field air blanks (n = 5) were conducted by placing a blank filter at the center of the sampling grid after the dryer cycle ended and leaving it exposed for one hour to test for atmospheric deposition when the dryer vent was not running and establish if the presence of “other’ fibers was a result of atmospheric deposition, the dryer’s emissions or a combination. Although fibers were observed in the field blanks (7 ± 4.3(SD)), their relatively low numbers are negligible when compared to the high numbers in the results. Furthermore, it is possible they were emitted from the vent but just took longer to settle after the dryer stopped running or might have been relocated by the wind. Filters were covered at all times during microscope inspection. Air blanks were collected when removing dryer exhaust lint from nylon mesh bags (n = 14). Filters were weighed before and after being exposed for the duration it took to thoroughly remove lint from the bag (5–10 minutes). The average number of fibers across blanks was 9.6±7.3(SD), and there was no weight change to the filter, deeming the number of contaminant fibers negligible. Furthermore, it is likely that these fibers came from the vent exhaust sample itself during removal from the bag, and not from surrounding air.

### Statistical methods

All statistical analyses and graphical displays were conducted in R [40] (R 4.0.0, R Core Team, 2020) with p< 0.05 indicating statistical significance. A Shapiro-Wilk test indicated that the number of pink fibers per snow surface samples were not normally distributed, therefore only nonparametric tests (e.g. Spearman's rank correlation, Kruskal-Wallace, Mann Whitney U) were used in the analysis.

## Results

### Surface snow sampling: Count

**Fig 5:**
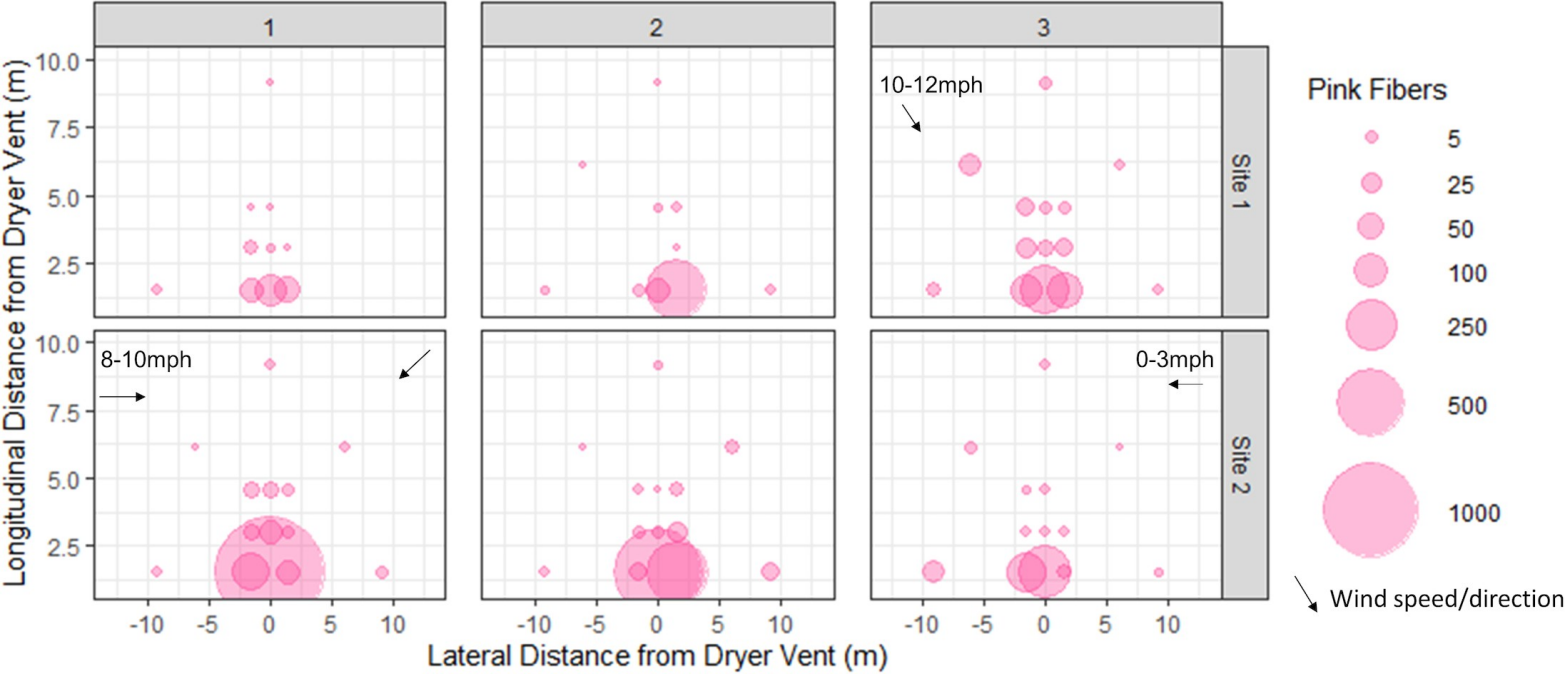
Pink fiber distribution for each dryer cycle at Site 1 (top row) and Site 2 (bottom row) locations. Wind speed and direction are indicated by black arrows, unless there was no wind detected/forecast on that sampling date. Plots in which no fibers were found are not shown.

Results from the two different locations and electric dryers varied in the number of fibers emitted, but showed similar patterns in terms of fiber distribution. At Site 1, the total number of pink fibers landing in all 14 sampling plots (0.093m^2^) per sampling event showed an average ± SD of 404 ± 192 (n = 3). The number of pink fibers observed in the individual plots ranged from 0–374, with a median of 4 an average ± SD of 29 ± 69 (n = 42) (Fig 5). At Site 2, the total number of pink fibers landing in all 14 sampling plots per sampling event showed an average ± SD of 1,169 ± 606 (n = 3). The number of pink fibers observed in the individual plots ranged from 0–1371, with a median value of 7 and an average ± SD of 85.5 ± 256 (n = 42) (Fig 5). A Mann Whitney U test revealed no statistical difference in the number of pink fibers counted between the two sites (W = 732, p = 0.13).

The large variation in these numbers is explained by noting that the majority of fibers (85.5% in Site 1 and 92.5% in Site 2) collected within the 3 plots were located closest to the vent (Fig 5). The number of pink fibers decreased with increased distance from the dryer vent (Spearmans’ Rank Correlation (r_s_ = -0.58, p < 0.05). A Kruskal Wallace test followed by a post-hoc analysis (Dunn’s Test) indicated that there were significant differences between the number of fibers counted in plots 5ft (1.52m) from the vent with those counted in plots at 10ft (3.05m), 15ft (4.57m) and 30ft (9.14m) (p<0.001). However, pink fibers were found in each of the fourteen 0.093m^2^ surface snow plots though not on every sampling date. Including both sites, an average ± SD of 5.43±8.24 (median = 3, range 0–36, n = 30) pink fibers were detected in snow samples 30ft (9.14m) from the vent, with a maximum of 29 (Site 1) and 36 (Site 2) counted in some of these furthest plots.

There appears to be a relationship between wind and fiber distribution, as demonstrated at Site 2. When the wind was blowing perpendicular to the vent (parallel to the house), the concentration of fibers increased in sample sites downwind of the vent, notably in the 5ft (1.52m) and 30ft (9.14m) plots of dry cycle 3 (Fig 5). When there was no wind or wind blowing back and forth, the fibers were distributed more symmetrically as noted in dry cycle 1 (Fig 5). Though no wind was felt during snow sample collection, our data suggest that there might have been some breeze blowing from right to left in Site 1 cycle 1 and left to right in Site 2 cycle 2 during the dry cycle.

**Fig 6:**
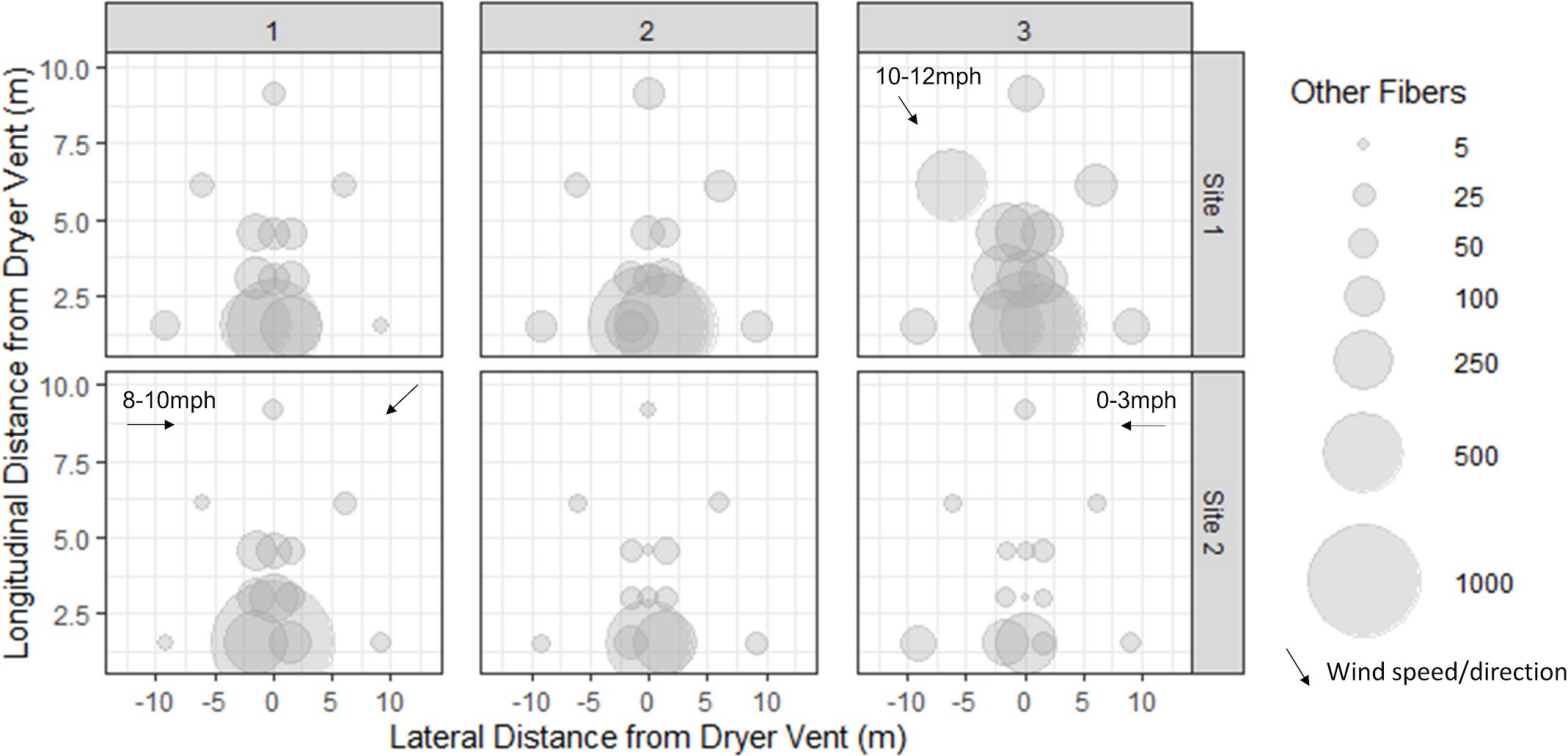
Distribution of other anthropogenic colored fibers (synthetic and non-synthetic) for each dryer cycle at Site 1 (top row) and Site 2 (bottom row) locations. Wind velocity and direction are indicated by black arrows, unless there was no wind detected/forecast on that sampling date.

Though this study’s aim was to investigate the distribution and total weight of fibers emitted into the air via dryer exhaust from a single pink polyester fleece blanket, pink fibers directly from the blanket only comprised an average of 11.8 ± 2% and 47 ± 7% of the total number of anthropogenic fibers collected from the snow samples at Sites 1 and 2, respectively. Therefore, it is important to note that other fibers microscopically identified as anthropogenic (Fig 3 and S1 File) were also observed in the snow plots. At Site 1, the total number of other anthropogenic fibers collected from all 14 sampling plots per sampling event showed an average ± SD of 2909 ± 1016 (n = 3), while the number of fibers collected from the individual plots ranged from 12–1144, with a median of 78 and an average ± SD of 208 ± 268 (n = 42) (Fig 6). At Site 2, the total number of other anthropogenic fibers collected from all 14 sampling plots per sampling event showed an average ± SD of 1330 ± 793 (n = 3), while the number of fibers collected from the individual plots ranged from 3–1226, with a median value of 23 and an average ± SD of 95 ± 209 (n = 42) (Fig 6).

For comparability with present and future studies, results are expressed in the number of fibers/m^2^ and in the number of fibers/L in Table 2. However, it is important to consider that the percent water content of snow varies greatly from day to day and location to location, causing fluctuations in the amount of meltwater from collected snow samples, and therefore reporting the number of fibers/L may not accurately represent fiber concentrations. In addition, our results show variation in the number of fibers collected at varying distances from the vent, thus one must be cautious in extrapolating these numbers to represent a larger surface area.

**Table 2:** Average ± SD of fibers observed in sampling plots expressed in different units for comparability with other studies.

Location	Fibers/plot (0.093m^2^)	Fibers/m^2^	Fibers/L
**Site 1**			
Pink Blanket Fibers	404 ± 192	4344 ± 2066	729 ± 439
Other fibers	2909 ± 1016	31283 ± 10924	7551 ± 1879
Total Fibers	3313 ± 1205	35627 ± 12953	8280 ± 2023
**Site 2**			
Pink Blanket Fibers	1,169 ± 606	12570 ± 6514	5065 ± 2775
Other fibers	1330 ± 793	14305 ± 8528	5673 ± 3492
Total Fibers	2499 ± 1356	26875 ± 14581	10738 ± 6154

### Dryer vent exhaust: Weight

**Fig 7:**
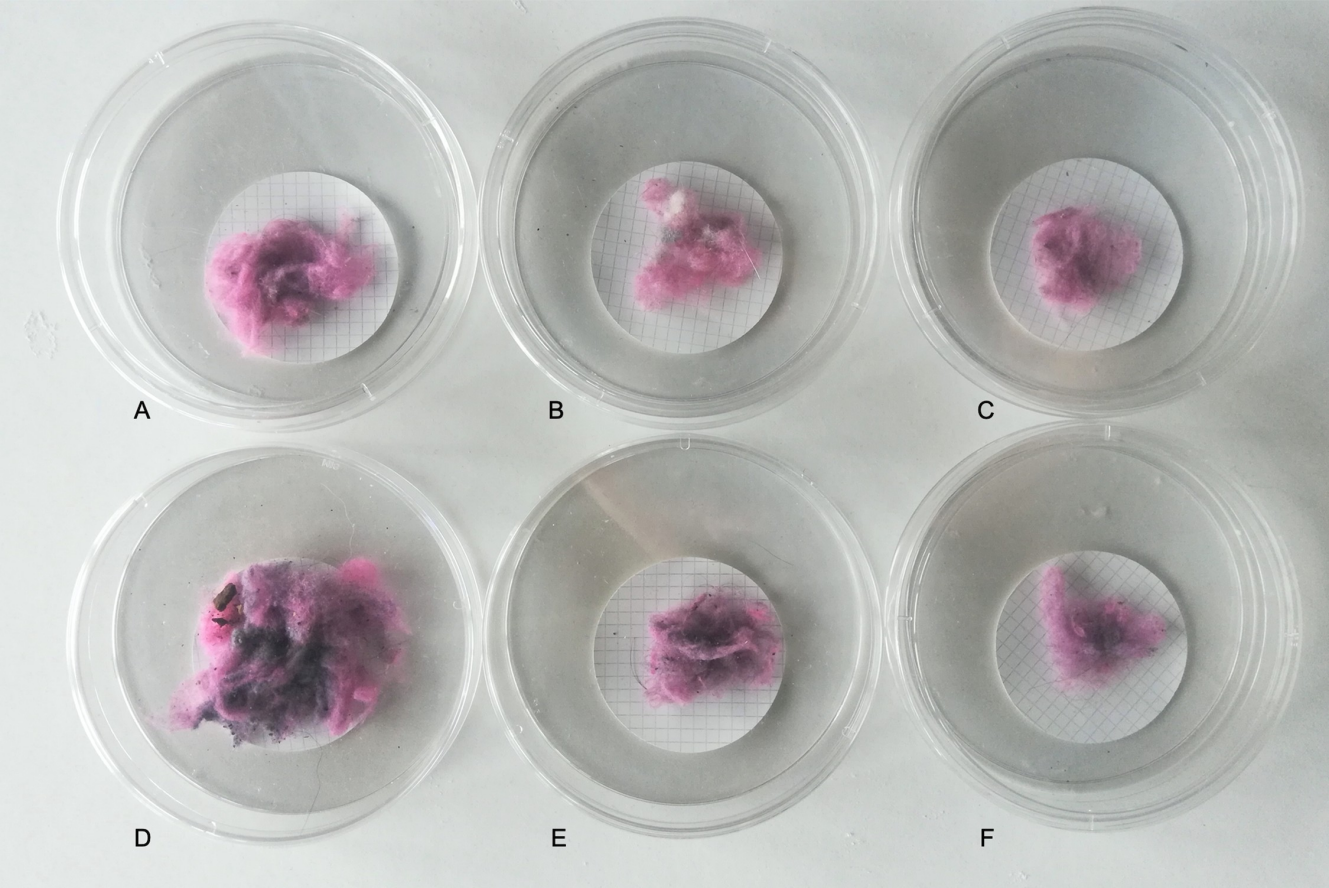
An example of lint collected in nylon mesh bags from vent exhaust for Site 1 dry cycle 1 (A), 2 (B) and 3 (C) and for Site 2 dry cycle 1 (D), 2 (E) and 3 (F).

**Fig 8:**
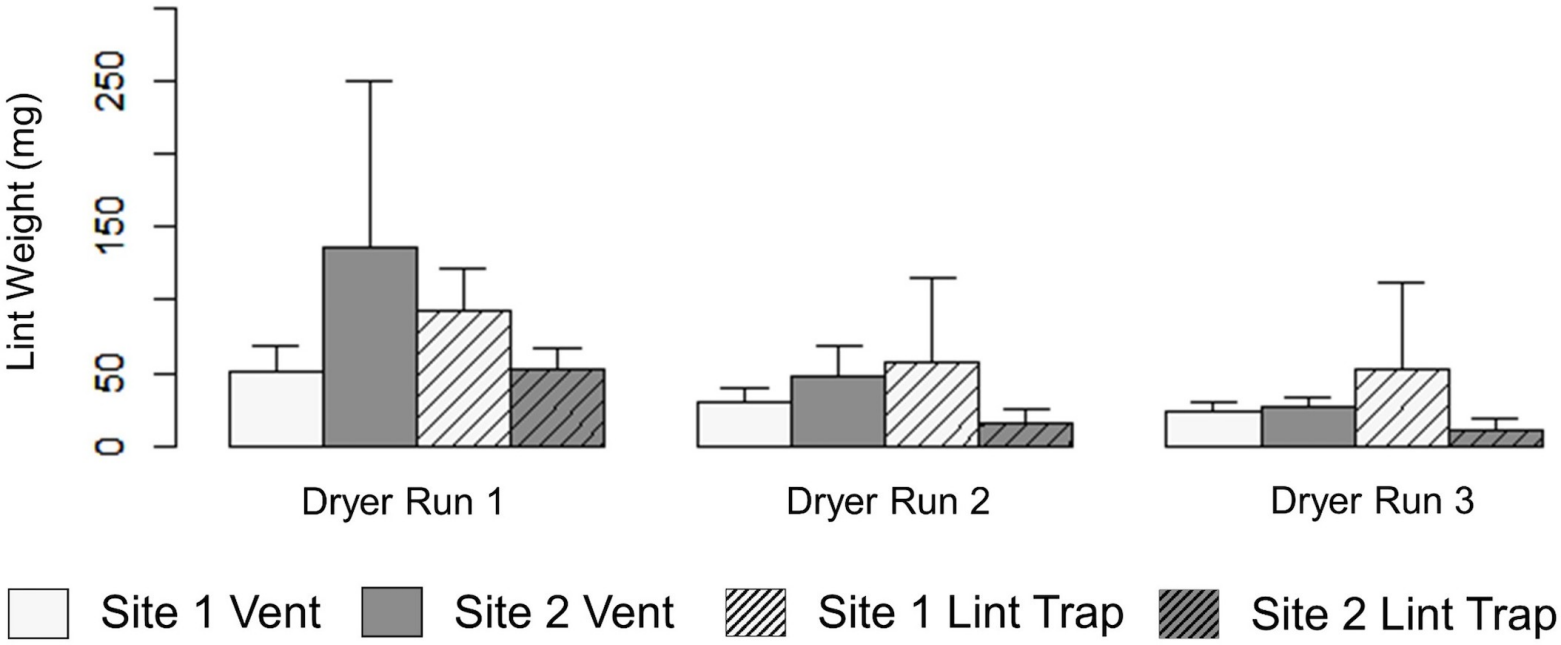
A comparison of mean weight (mg) (n = 3) of lint captured via dryer vent exhaust and in dryer lint trap per dry cycle at the two sites (error bars represent standard deviation).

**Fig 9:**
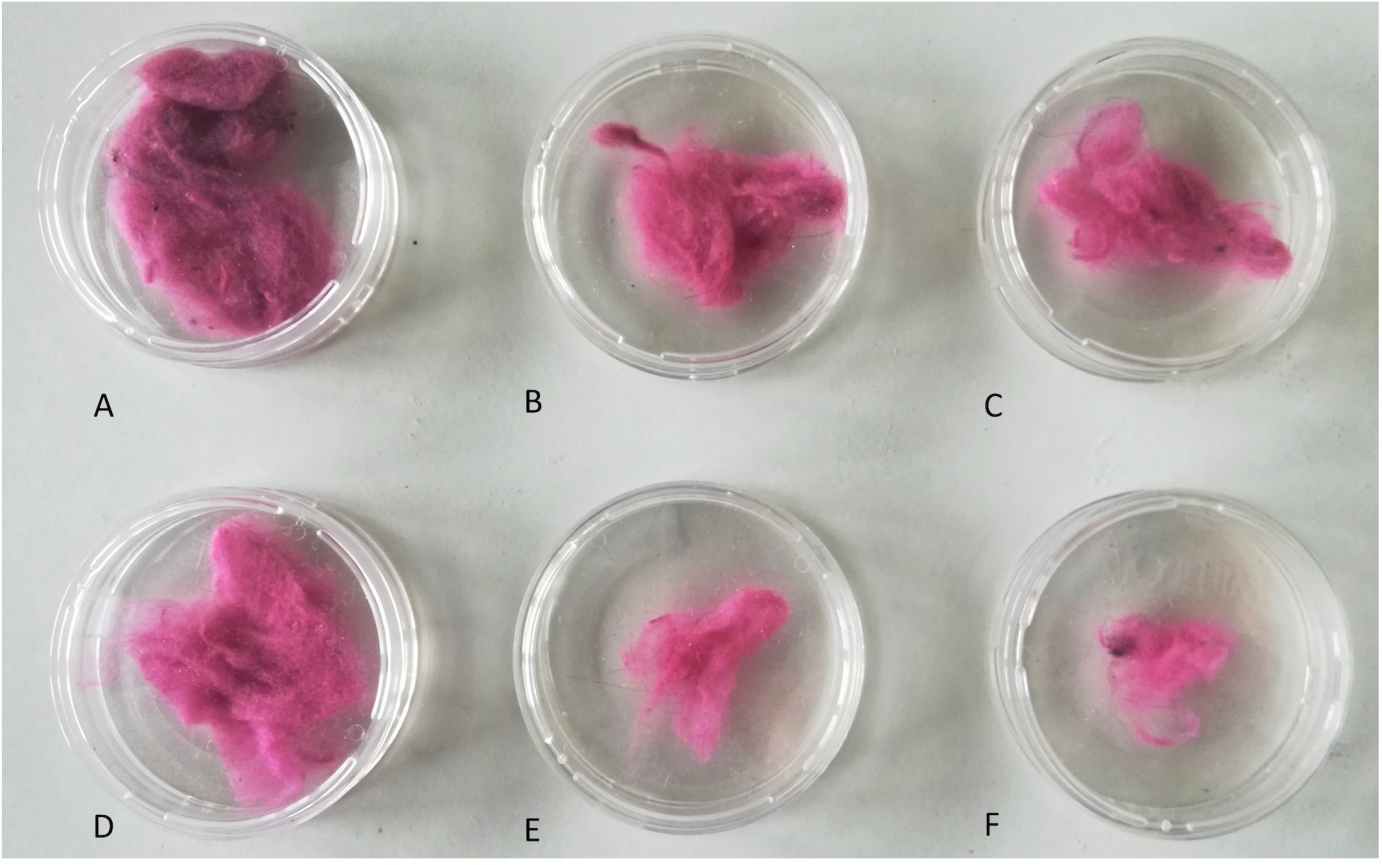
Examples of lint captured in dryer lint traps from Site 1 (A = dry cycle 1, B = dry cycle 2, C = dry cycle 3) and from Site 2 (D = dry cycle 1, E = dry cycle 2, F = dry cycle 3).

On average, the weight (mg) of lint emitted via vent exhaust for all dryer cycles and replicates (n = 9) from Site 1 (35 ± 16mg) was less than that of Site 2 (70 ± 77mg) (Figs 7 and 8). These results are consistent with the snow sampling results in which more fibers were observed in the plots at Site 2 than at Site 1 (Figs 7 and 8). Furthermore, the amount of lint captured in vent exhaust from the first dryer cycle to the third significantly decreased at each location and in each replicate (Kruskal Wallace, p<0.007) (Figs 7 and 8). Interestingly, the lint trap at Site 1 collected more lint on average (68±47(SD)mg) than Site 2 (27± 22(SD)mg) (Figs 8 and 9).

## Discussion

**Fig 10:**
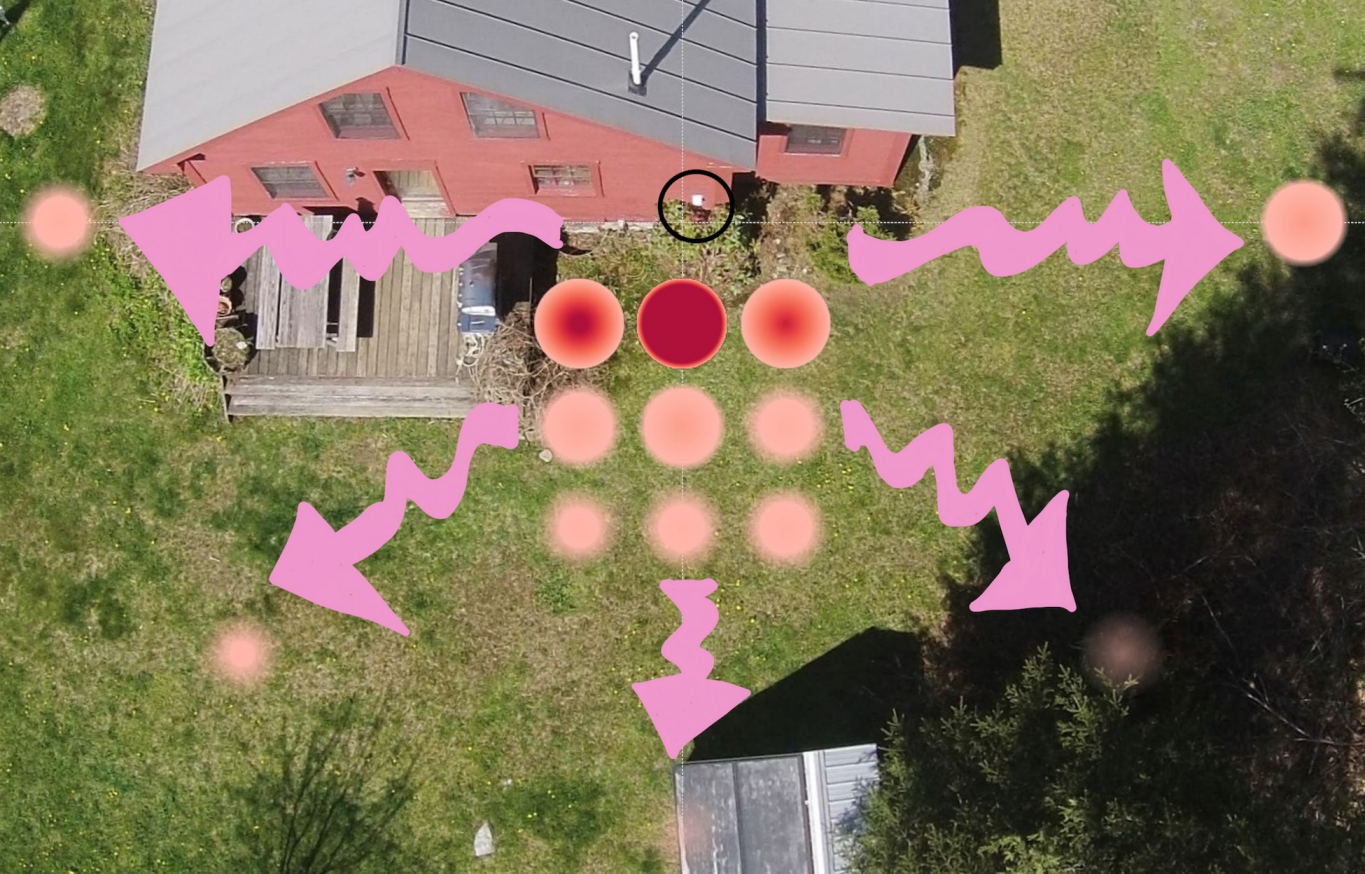
Accumulation of pink fibers from all three dryer cycles at site 2.

Results from the snow and dryer exhaust samples in this study demonstrate that anthropogenic fibers from textiles, regardless of type, escape the dryer lint trap and are emitted into the air via dryer vent exhaust. Further study is needed to determine the fate of these fibers once they enter the environment, since this study indicates that fibers can travel at least 30ft (9.14m) from the vent itself (Fig 10). Our results suggest that wind may be an important factor in determining fiber deposition patterns from dryer vents. With additional sampling in which wind direction and velocity are tested variables, results could predict transportation patterns and demonstrate that fibers are potentially transported further into the air and deposited at greater distances, therefore being an important source of atmospheric fiber pollution. Investigating the distribution and travel pathways of microfibers once they leave a dryer vent that specifically addresses how far fibers ultimately travel, the mechanisms of transport (such as wind, rain, runoff, animals), and if fiber characteristics (e.g. fiber length) influence transport will enhance our understanding of dryers as a source of microfiber pollution.

This study establishes that there are variations in microfiber emissions between different electric clothes dryers. The variation in the number of fibers counted in the snow sampling plots and captured from dryer exhaust at the two sites could be a factor of dryer design, age, installation and lint trap characteristics. Not only was a greater weight of material emitted from the dryer vent at Site 2, but the dryer at Site 1 consistently collected more lint. This may be due to differences in the lint trap design of each dryer. For example, the surface area of the lint trap at Site 1 (851cm^2^) was approximately three times greater than that of Site 2 (282cm^2^). Also, the ducting at Site 1 was rigid, approximately 3 times longer and at least 10 times older than that of Site 2. Fibers build up over time increasing resistance and friction within the duct, potentially trapping fibers in the ducting between the dryer drum and vent. Investigating how different types and sizes of dryers, dryer power and air flow, lint trap design, dimensions and shape of ducting, ducting pathways and cleanliness, vent design, vent covers and vent height, dryer settings, age and use affect the presence of built up fiber and fiber emissions will be beneficial in understanding microfiber pollution caused by electric clothes dryers and help to inform design and implementation strategies that could reduce microfiber pollution.

Understanding the extent to which electric clothes dryers contribute to microfiber pollution will be important in understanding the comparative effect washing versus drying has on the breakdown of textiles while providing a more comprehensive understanding of the impacts that washing and drying laundry has on the environment. Results from this dryer study and results from washing machine studies show that the loss of microfibers decreases with each consecutive cycle [19, 20]. Investigating the shed pattern and rate over a textile’s life would help estimate the total amount of microfiber emitted by dryer exhaust. In addition, future studies addressing how washing machine use before drying affects the amount of microfibers released via dryer vent exhaust (whether washing increases or reduces the likelihood of shedding in the dryer or has no effect), will help to inform ways to reduce microfiber pollution. This study does not address alternative forms of drying. Investigating whether alternative methods of drying such as in air (in drying closets and on lines outside), condenser dryers, combined washer/dryers and gas dryers which also cause microfiber shedding and release into the environment, will help to identify alternative drying methods that potentially reduce microfiber emissions. Furthermore, in order to generate a robust value for the total environmental emissions caused by laundry: using washers plus electric dryers (or drying clothing in general), it must be acknowledged that washing machine effluent undergoes some form of waste water treatment before possibly entering the environment, while dryer exhaust is released directly into the atmosphere.

## Conclusion

The goal of this study was to determine if and to what extent dryers are a source of microfiber pollution in the environment. Our results establish that electric clothes dryers are contributing a potentially large volume of synthetic and non-synthetic microfibers from clothing and home textiles into our environment, demonstrating a need to develop and implement strategies/equipment that reduce microfiber pollution from dryers. Potential solutions driven by this study coupled with further investigations (described above) should address both the equipment itself and consumer behavior. This study suggests that household dryers must be included in the discussion alongside household washing machines when considering strategies, policies and innovations to prevent and mitigate microfiber pollution.

## Supporting information

S1 File Additional images detailing methods and results. (DOCX)

S1 Dataset Raw data for snow sampling plots, dryer exhaust lint weight and blanks at Site 1 and 2. (XLSX)
