# Supplementary material for: Electric clothes dryers: An underestimated source of microfiber pollution
Source: PLoS One. 2020 Oct 7;15(10):e0239165. doi: 10.1371/journal.pone.0239165 (PMC7540867; doi:10.1371/journal.pone.0239165)
Supplement: S1 File — (DOCX) [file pone.0239165.s001.docx]

**S1 File. Additional images detailing methods and results.**


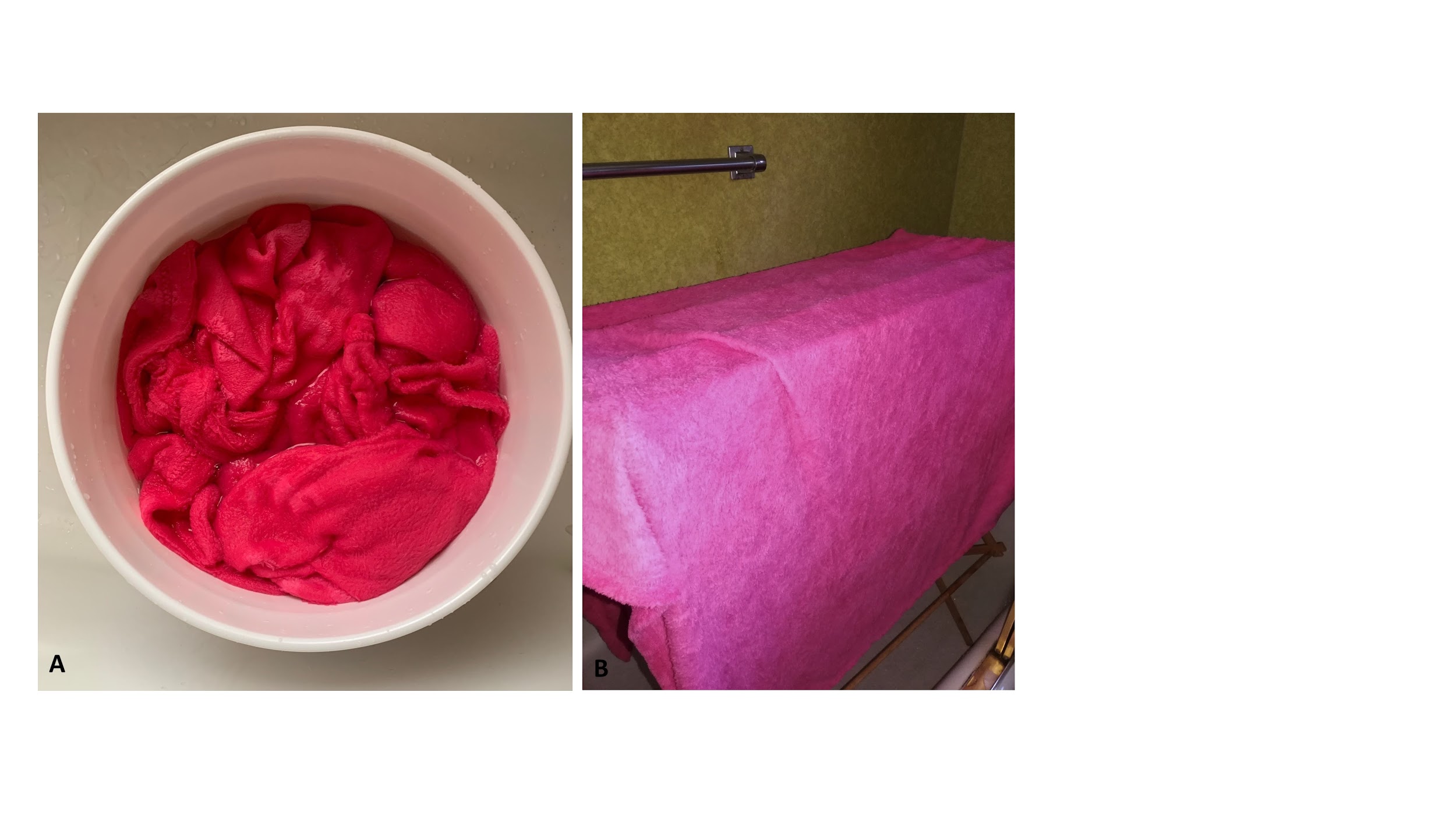


**S1 Fig 1**. Blanket soaking for 5 minutes in 5-gallon bucket (A) and

hang drying for 30 minutes (B).


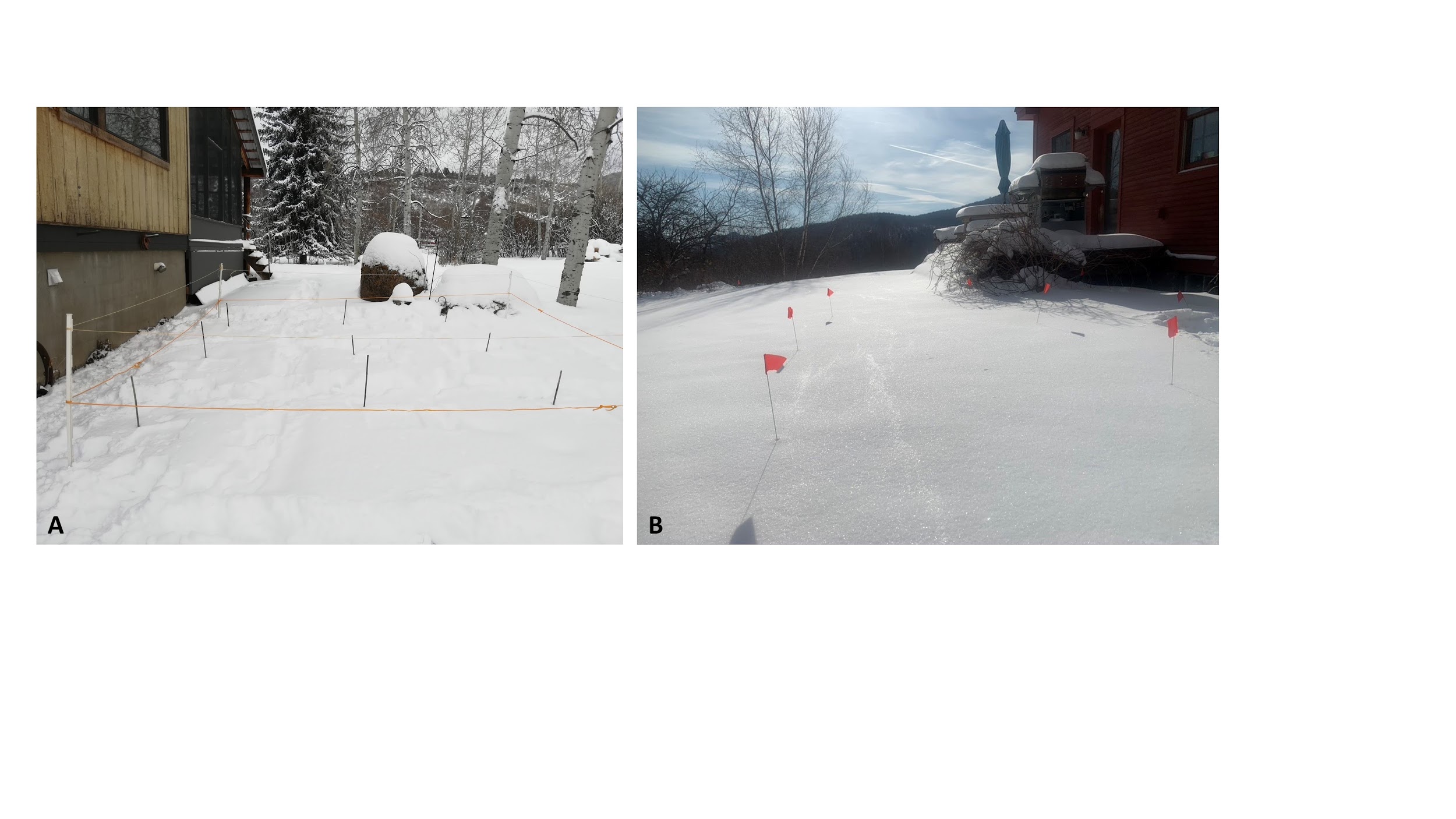


**S1 Fig 2**. Examples of marked snow sampling plots at Site 1 (A) and Site 2 (B).


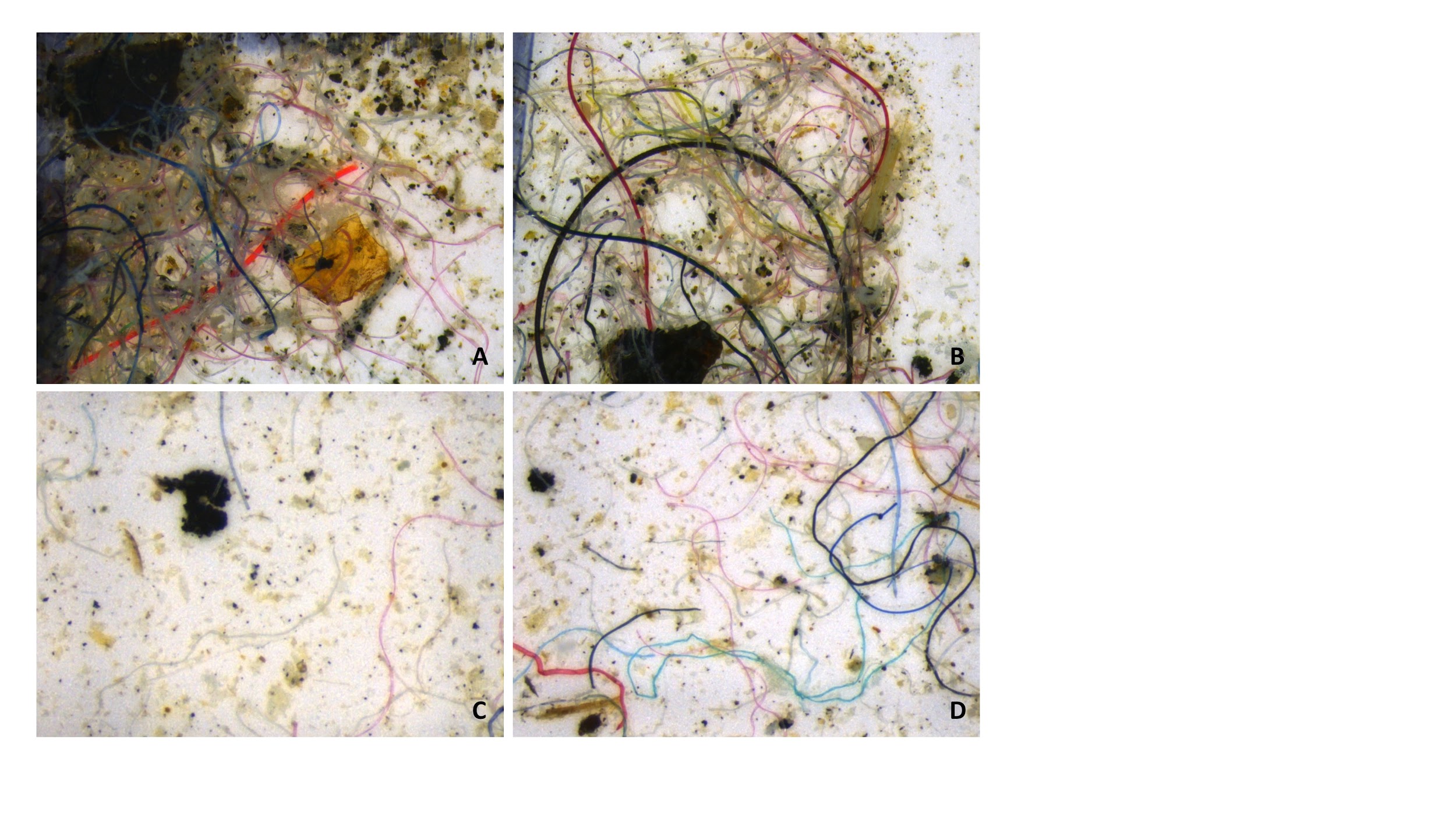


**S3 Fig**. Examples of all fibers (non-synthetic and synthetic) observed in

snow samples (images from plots at Site 1,1.524m from vent).

**S1 Table 1**. Weight (mg) of lint including fibers captured in dryer vent exhaust.


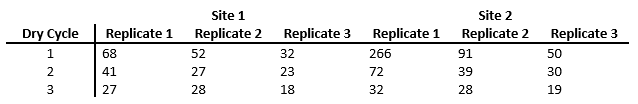


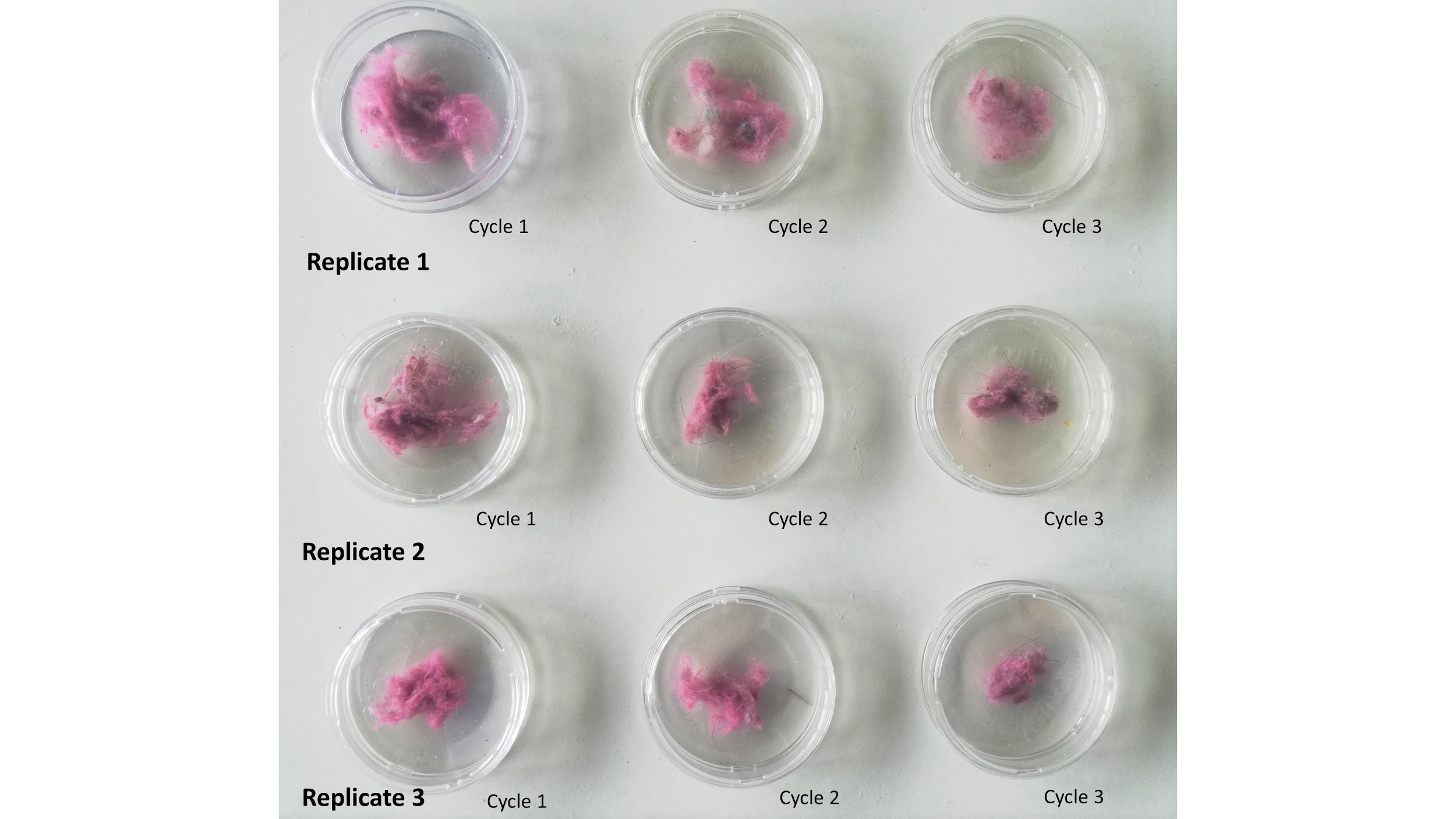


**S1 Fig 4.** Lint collected in nylon mesh bags from dryer vent exhaust at

Site 1 for each replicate and dry cycle.


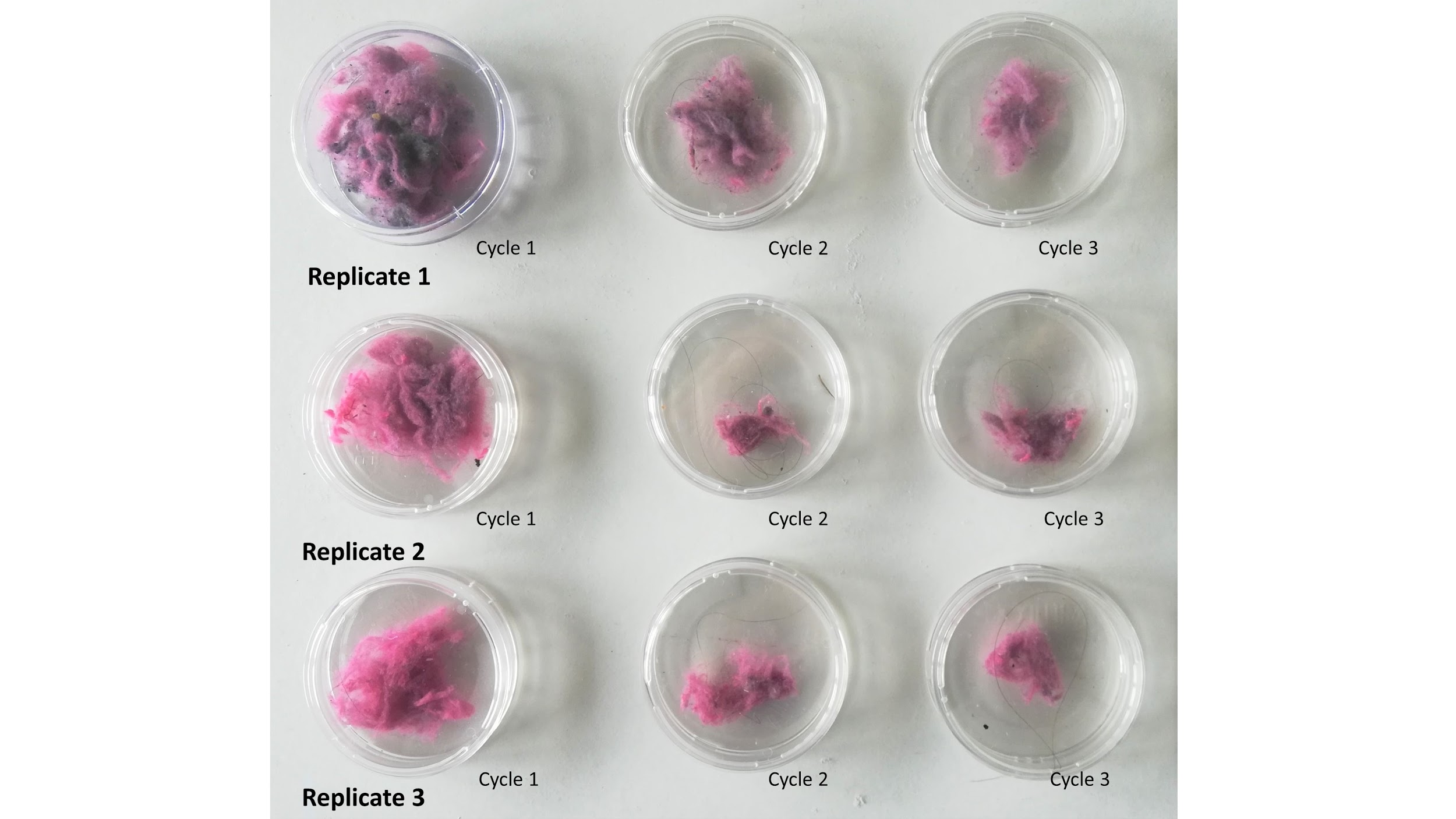


**S1 Fig 5**. Lint collected in nylon mesh bags from dryer vent exhaust

Site 2 for each replicate and dry cycle.


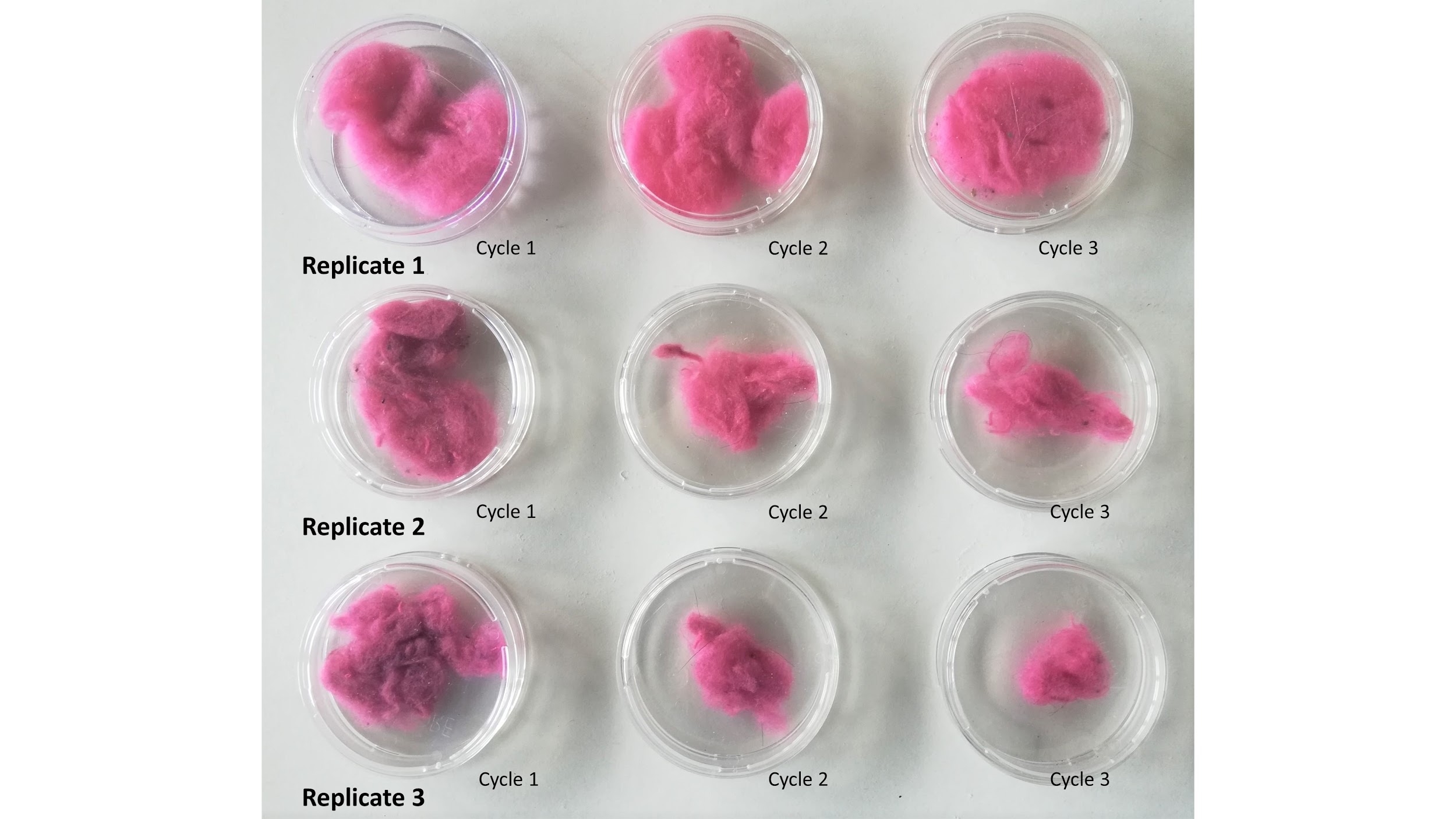


**S1 Fig 6.** Lint collected from dryer lint traps at Site 1 for each bag

replicate (corresponding to Image S6).


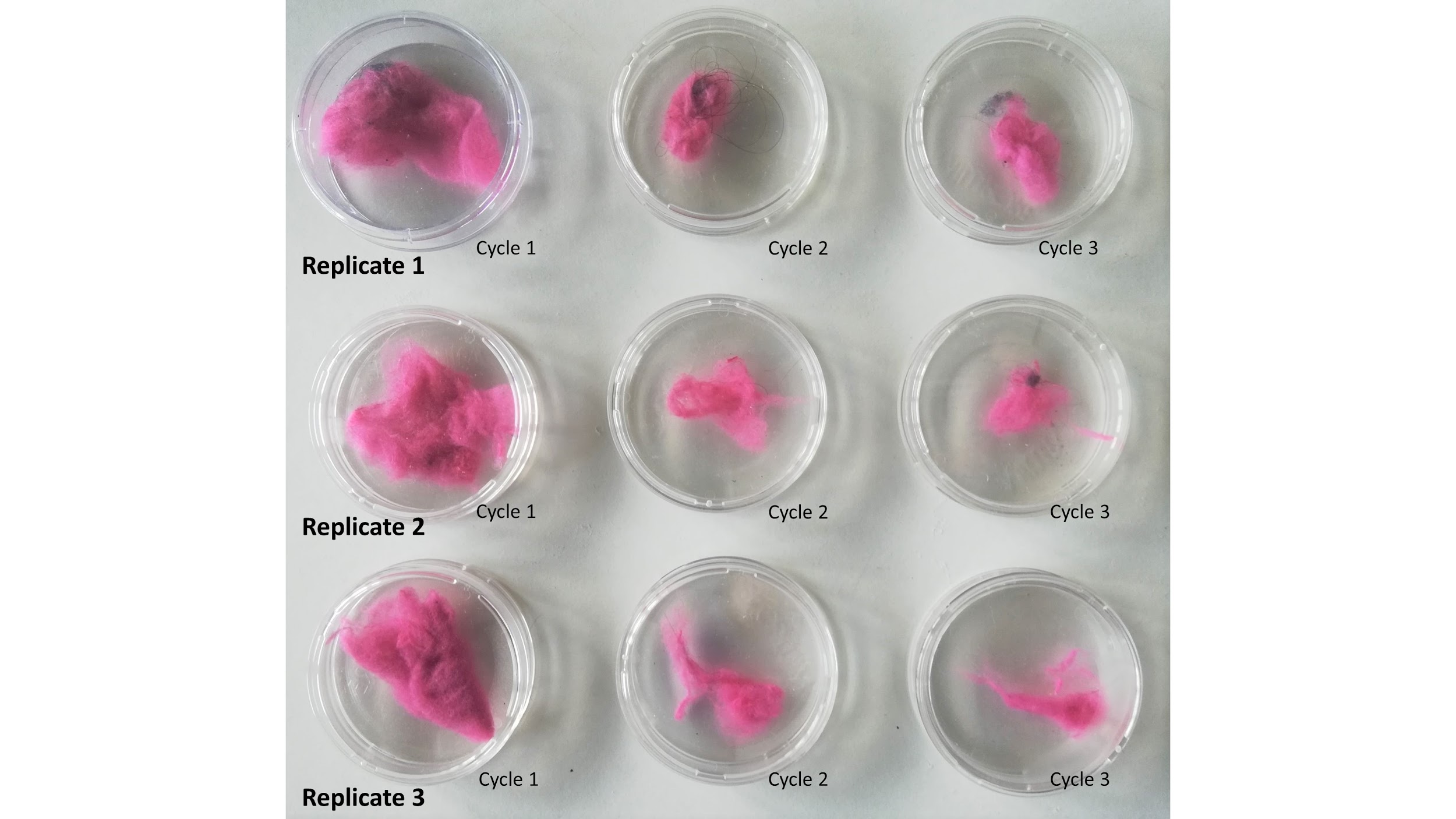


**S1 Fig 7.** Lint collected from dryer lint trap at Site 2 for each replicate and dry cycle (corresponding to Image S7).


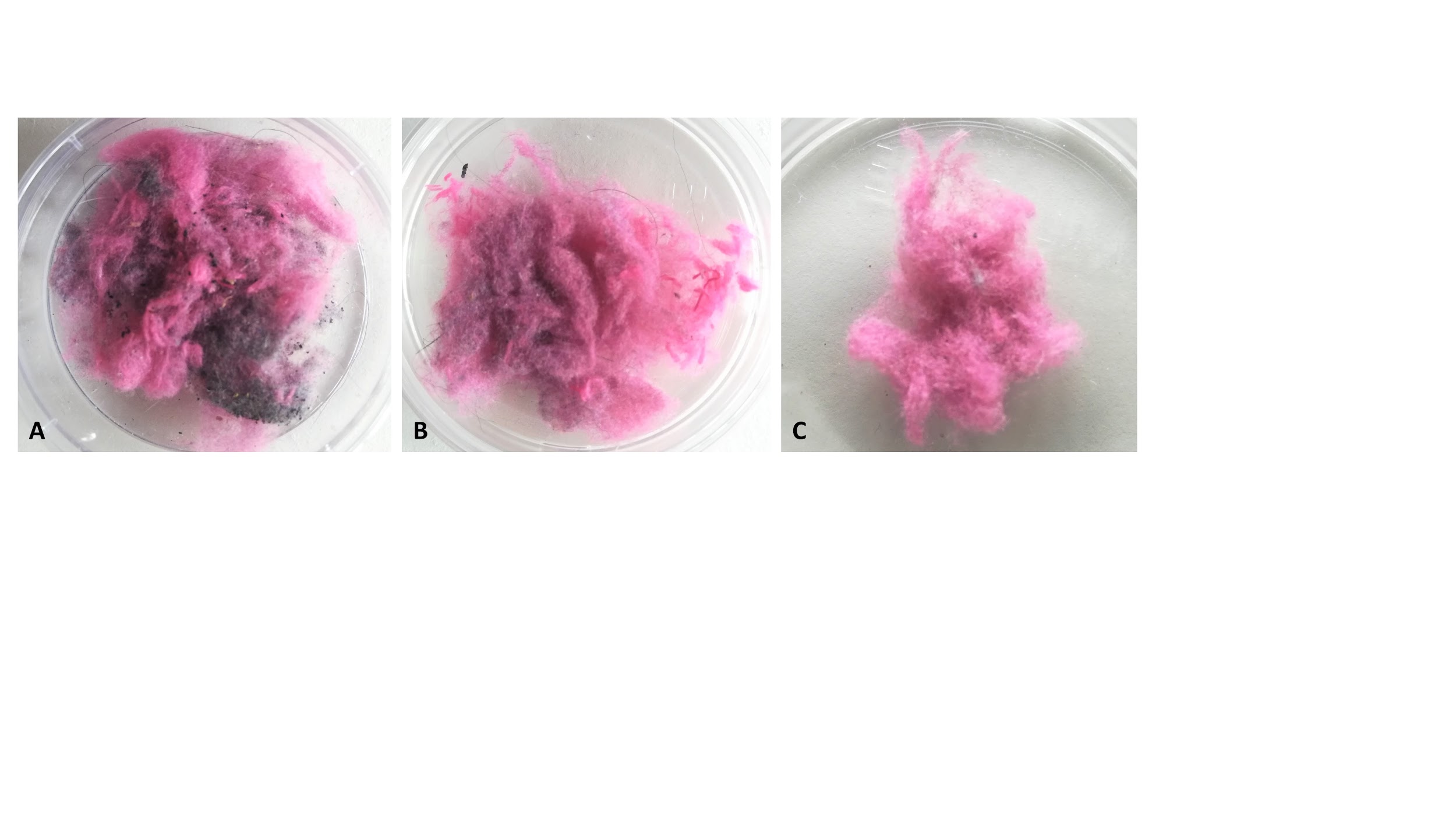


**S1 Fig 8.** Lint collected from dryer vent exhaust from first dry cycle A) Site 2, 266mg B) Site 2, 91mg C) Site 1, 32mg, including other fibers mixed in with polyester fleece fibers.
